# Reducing Maternal Deaths in Ethiopia: Results of an Intervention Programme in Southwest Ethiopia

**DOI:** 10.1371/journal.pone.0169304

**Published:** 2017-01-03

**Authors:** Bernt Lindtjørn, Demissew Mitiku, Zillo Zidda, Yaliso Yaya

**Affiliations:** 1 Centre for International Health, University of Bergen, Bergen, Norway; 2 Gidole Hospital, Gidole, Ethiopia; 3 Arba Minch Hospital, Arba Minch, Ethiopia; Stellenbosch University, SOUTH AFRICA

## Abstract

**Background:**

In a large population in Southwest Ethiopia (population 700,000), we carried out a complex set of interventions with the aim of reducing maternal mortality. This study evaluated the effects of several coordinated interventions to help improve effective coverage and reduce maternal deaths. Together with the Ministry of Health in Ethiopia, we designed a project to strengthen the health-care system. A particular emphasis was given to upgrade existing institutions so that they could carry out Basic (BEmOC) and Comprehensive Emergency Obstetric Care (CEmOC). Health institutions were upgraded by training non-clinical physicians and midwives by providing the institutions with essential and basic equipment, and by regular monitoring and supervision by staff competent in emergency obstetric work.

**Results:**

In this implementation study, the maternal mortality ratio (MMR) was the primary outcome. The study was carried out from 2010 to 2013 in three districts, and we registered 38,312 births. The MMR declined by 64% during the intervention period from 477 to 219 deaths per 100,000 live births (OR 0.46; 95% CI 0.24–0.88). The decline in MMR was higher for the districts with CEmOC, while the mean number of antenatal visits for each woman was 2.6 (Inter Quartile Range 2–4). The percentage of pregnant women who attended four or more antenatal controls increased by 20%, with the number of women who delivered at home declining by 10.5% (P<0.001). Similarly, the number of deliveries at health posts, health centres and hospitals increased, and we observed a decline in the use of traditional birth attendants. Households living near to all-weather roads had lower maternal mortality rates (MMR 220) compared with households without roads (MMR 598; OR 2.72 (95% CI 1.61–4.61)).

**Conclusions:**

Our results show that it is possible to achieve substantial reductions in maternal mortality rates over a short period of time if the effective coverage of well-known interventions is implemented.

## Introduction

Global maternal mortality rates were halved between 1990 and 2010. However, most of the maternal deaths in the world occur in developing countries [1]. Earlier studies have shown that maternal health services do not reach those who most need them, and the quality of obstetric services has been poor [2, 3]. To help address these disparities and speed up progress in reducing maternal deaths, the World Health Organization (WHO) and national governments have made health-care system strengthening a key priority for improving maternal and child health and access to health care [2, 4].

Previous studies have documented that health-care system weaknesses can create barriers to the effective use of prevention and care. Such barriers may include physical access, poverty, inadequate staffing and unnecessary delays. However, to improve access, utilization alone is insufficient. The WHO recommends combining need, use and quality of care as ‘‘effective coverage”: “The proportion of a population that needs a procedure that actually receives it” [5].

We carried out a health-care system strengthening intervention in rural Southwest Ethiopia designed to reduce maternal deaths. The main strategy was to improve an effective coverage of Comprehensive Emergency Obstetric Care (CEmOC) and Basic Emergency Obstetric Care (BEmOC). Our intervention focused on strengthening existing institutions in rural areas, increasing the capacity and quality of work at health institutions and increasing referrals to hospitals through the work of Health Extension Workers (HEW) and health centres. We strengthened the clinical infrastructure by training non-physician clinicians to carry out CEmOC [6], thereby improving the essential skills of midwives and nurses for BEmOC [7] by equipping institutions with essential instruments to do basic and comprehensive emergency procedures, and by establishing a system of close supervision and monitoring. The main tool for monitoring was through a birth registration at three districts with a population of 700,000 [8].

The maternal mortality ratio (MMR) was the conventional key indicator to help monitor progress towards the Millennium Development Goal 5 (MDG5) target of reducing it by 75% by 2015 from the level in 1990 [9]. However, measuring maternal mortality is difficult to quantify in developing countries because of a limited registration in births and deaths [10]. In addition, the rate of reduction in maternal mortality rates was slower than expected [11]. In 2013, UNICEF reported that 44% of births in sub-Saharan Africa, but only 7% in Ethiopia, were registered [12]. Although indirect methods such as the modelling of proxy data (for example sisterhood methods) provide important information for global and national planning, they have a limited use in monitoring progress in reducing maternal deaths at the district level. In our programme to reduce maternal deaths in Southwest Ethiopia, we developed and validated a population-based birth registry to monitor the progress of the interventions to reduce maternal and neonatal deaths [8].

The aim of this study is to describe how the implementation of interventions such as training, equipping institutions, in addition to the monitoring and supervision that took place in the area over a six-year period from 2008 to 2013, can reduce maternal mortality rates.

## Methods

### Study setting

This implementation study aimed at reducing maternal deaths analyses the outcomes of interventions done in three of the districts (locally called woreda) in Southwest Ethiopia (the Arba Minch Zuria, Bonke and Dirashe woredas), which had a monitoring system to measure maternal deaths. It was done during the first years of the Ethiopian Health Sector Strategic plan (2010–2015), which emphasized improving maternal and neonatal health [13].

The new Ethiopian health tier system consists of primary level health-care units (primary hospitals that serve a population of 60,000 to 100,000, health centres that serve a population of 15,000 to 25,000 and health posts that serve a population of 3,000 to 5,000 people), as well as secondary level health care including a general hospital (catchment area of 1,000,000 to 1,500,000 people) and tertiary level specialized hospitals, which serve 3.5–5 million people [14]. Most often, the primary health-care unit consists of a health centre and five satellite health posts. Health posts serve as treatment places and are found in kebeles with an average population of 5,000 people. A health post is staffed by HEWs, and they work on health promotion and disease prevention through regular home visits in their catchment area [14, 15]. In addition, they give a prioritized follow-up to households with pregnant women, new-born babies and sick people. HEWs also provide antenatal examinations and delivery services, both at home and health posts. In addition, five to 10 laywomen, known as volunteer health promoters, assist the HEWs, and in 2013 approximately 38,000 health extension workers served villages (kebeles) in the country.

Health centres provide curative and preventive services for approximately 25,000 people, with a staff composition of health officers (people with four-year clinical and preventive health education at a university), nurses, midwives, laboratory technicians and pharmacy technicians. Hospitals have medical doctors, in addition to other professionals with the specialty depending on the status of the hospital.

In our area of working, we used the skills of these workers to register births and maternal deaths, and also to monitor whether there was a decline in maternal and neonatal deaths during our intervention [16]. During this period, the government also discussed the need to set up vital registration systems, including birth registration and the Central Statistical Agency suggested, thereby that the HEWs found in all local communities could be responsible for such registration. We therefore set up, field tested and implemented a pilot scheme to register births and neonatal deaths [8, 16]. Simultaneously, the WHO started with the maternal death review and maternal death surveillance and response guidelines as part of an international effort to monitor the work to reduce maternal deaths [17]. The “Maternal Death Surveillance and Response” was also started in Ethiopia, but was implemented in our study area fairly late [14].

### Study population

**Fig 1:**
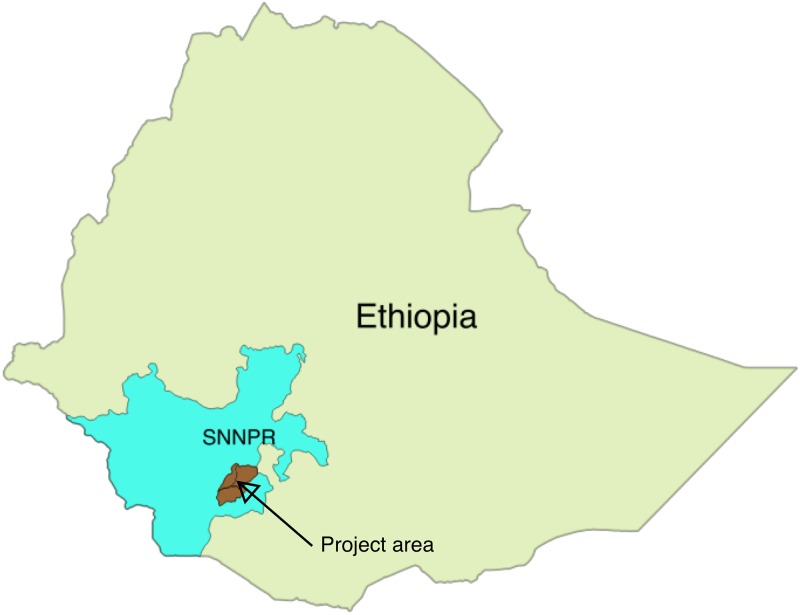
Map of project area. SNNRP: Southern Nations, Nationalities and Peoples' Region.

This intervention study was done in the three woredas (districts) of Arba Minch Zuria, Bonke and Dirashe) in two zones (the Gamo Gofa and Segen Areas Peoples’ Zones) in South-west Ethiopia in the Southern Nations, Nationalities and Peoples' Region (see Fig 1). Our intervention area represents three climatic areas (cold, temperate and hot) and most of the people live in the highlands 2,000 metres above sea level and practice subsistence farming. There are few all-weather roads, and many people live in areas without access to roads.

The population of the Gamo Gofa Zone was roughly 1.75 million people in 2010. The capital of the zone is Arba Minch Town, which is located approximately 500 km from Addis Ababa. Moreover, the population of the Segen Area Peoples’ Zone was 640,000 people in 2010.

In 2010, about 170,000 people lived in Bonke. This woreda (district) had no hospital providing CEmOC during the study; hence, people in need of such services had to travel to the nearest hospital, the Arba Minch Hospital, located approximately 100 kilometres from Bonke. In late 2014, a health centre in this area started to provide the population with comprehensive emergency obstetric care. In addition, the Ministry of Health started to build a hospital in the area. At the time of our study, approximately 380,000 people lived in the Arba Minch Zuria Woreda, and the district had one large hospital. The larger parts of the population in this district live in the Gamo highlands, far from this hospital. The people living here have limited road access for comprehensive delivery services. In 2010, roughly 142,000 people lived in Dirashe. This district is served by Gidole Hospital, which has a well-functioning maternity waiting area where mothers with high-risk pregnancies are referred to- and observed until delivery [18].

### Study design

This implementation study used the following outcome measures: maternal deaths measured as the maternal mortality ratio. The secondary outcomes were the proportion of skilled birth attendance, proportion of institutional deliveries, referrals to health posts, health centres or hospitals. We also measured the use of antenatal control. Explanatory variables included distance to institution, literacy of `both the husband and of delivering mothers, history of previous pregnancies and deliveries, and whether any illness had occurred during the pregnancy.

Because the interventions we used are believed to be effective [19], we considered it unethical to introduce a control area without access to such interventions. Our study analyses trended toward the use of interventions and simultaneously occurring improvements in outcome measures, especially maternal mortality.

### Procedures and description of health-care system strengthening interventions

Together with the Ministry of Health, we designed a project to strengthen the health-care system. A particular emphasis was given to upgrading existing institutions so that they could carry out BEmOC and CEmOC. These health institutions were upgraded by training non-clinical physicians and midwives by providing the institutions with essential and basic equipment, and by regular monitoring and supervision by staff competent in emergency obstetric work.

The aim of the project was to assure that each health facility had obstetric services available 24 hours a day and seven days a week, and were staffed by skilled health professionals. An Emergency Obstetric Care (EmOC) facility refers to whether or not an institution is fully functioning as a basic (BEmOC) or comprehensive (CEmOC) facility [4]. We defined the functioning by nine signal functions: administering parenteral antibiotics, administering parenteral oxytocic drugs, administering parenteral sedatives, manual removal of the placenta, removal of retained products of conception, assisted vaginal delivery (vacuum or forceps delivery) (BEmOC). Institutions, which in addition to these signal functions could do caesarean sections and have a blood transfusion service, were defined as CEmOC facilities.

At the start of our intervention, we conducted a review of the basic signal functions at three hospitals and 63 health centres in the Gamo Gofa Zone [3]. Our study showed that the availability, use and quality of BEmOC and CEmOC facilities fell below the accepted WHO standards [3]. The results of this mapping was used to strengthen the institutions, and a study done some years after this mapping showed that institutions had functional comprehensive or basic emergency services [20].

Each of the institutions were regularly supervised, at least once every quarter, to monitor the progress of the work. A specific emphasis was given to ensure that each institution practised the signal functions listed above, and to discuss how to deal with problematic deliveries and operations.

We actively used the delivery and operations registration books to conduct systematic audits of the work the previous months, and thus identify challenges and improve the services. Consequently, the supervisory visits also had the form of audits to monitor the work at the delivery units. Every delivery unit at an institution was led by a case-management team, and the supervisory visits took place at least once every quarter. In case unexpected incidences or complications had occurred, we evaluated events that had taken place using a no-blame culture [21, 22]. And events occurring at one institution were shared with staff from the other institutions, thereby promoting a continuous learning attitude among the staff [23]. Our experiences agree with the experience from countries such as the UK, which show that unit-based incident reporting, instead of institutional reporting systems, forms a better learning environment to improve services [24].

Table 1 outlines the provinces (woreda) we worked in, and describes the type of institutions and the work that they are supposed to do. Each health centre is responsible for at least five rural villages (kebeles). Two health extension workers serve each kebele, and each of the health extension workers received specific training on safe and clean delivery, and about when to refer patients to health centres or hospitals.

**Table 1:** Overview of hospitals and health centres in the intervention areas.

Province and type of institutions	Functions	2008	2013
**Arba Minch Zuria (Population 380,000 people)**			
** Hospitals**	**CEmOC function**	1	1
	**Blood bank**	1	1
	**Neonatal intensive care unit (NICU)**	0	1
** Health Centres with BEmOC functions**		0	5
**Bonke Woreda (Population 170,000 people)**			
** Hospitals**		0	0
** Health Centre with CEmOC function**		0	0
** Health Centres with BEmOC function**		0	2
**Dirashe Woreda (Population 142,000 people)**			
** Hospital**	**CEmOC**	1	1
	**Blood Bank**	0	1
	**NICU**	0	1
** Health Centres**	**CEmOC**	0	0
	**BEmOC**	0	6
	**Neonatal Care Unit**	0	6

Note—BEmOC: Basic Emergency Obstetric Care; CEmOC: Comprehensive Emergency Obstetric Care.

Parallel to the health interventions, we also set up a monitoring system to evaluate whether maternal mortality declined, if the mothers were referred or not, where the babies were delivered and who helped the mother during the delivery. We therefore set up a population-based birth registration system, and this has been validated and described in details [8]. The validation included a comparison of community-based birth registration with the sisterhood method, cross-sectional community surveys and with the institutional-based registration of maternal deaths [16]. Our conclusion is that the birth registration done by health extension workers provides a valid, community-based measurement of maternal deaths [16].

#### Assessment of quality of the training of non-clinical physicians

In 2009, during the second year of our work, we evaluated the results of the performance of non-clinical physicians and general practitioners doing Caesarean sections (C/S). This was done by prospectively recording operations done at the government hospitals and health centres. We reviewed the results of the health officers (non-clinical physicians with a BSc degree) and general practitioners who had been trained through this intervention programme at the Arba Minch Hospital. The assessment was done after they had completed a four-month training period. We studied all women undergoing a Caesarean section during the study period, and we recorded information on admission diagnosis, reason for surgery, preoperative conditions, with the outcome measures including neonatal and maternal conditions, post-operative complications and mortality.

During 2009, we collected data from 207 consecutive obstetric surgeries in four district health institutions in southern Ethiopia. These operations were done by general practitioners (38 operations; 18%) or health officers (169 operations, 82%). The main indications for surgery were cephalopelvic disproportion or obstructed labour (76 patients; 37%), foetal distress (43 patients; 21%), failure to progress (22 patients; 11%), previous C/S (13 patients; 6%), antepartum haemorrhage (12 patients; 6%), whereas the remaining operations were for uterus rupture, cord prolapse, breech in primigravidae and eclampsia. A Caesarean section (C/S) was done on 199 operations, and eight operations were for uterine rupture.

Two mothers (0.9%) died during their operation or during their stay at the hospital. There were 23 (11%) stillbirths, and five children (2%) died during the stay at the hospital. The rate of stillbirths varied among the hospitals, with the highest rate being at the newly started hospital in Saula (17 of 23 stillbirths and three out of five early neonatal deaths). Intraoperative complications occurred in five patients. Three of them were serious. and one re-operation was done because of bleeding. Three patients needed post-operative blood transfusions, and six patients had post-operative infections. Lastly, the mean duration of stay was seven days after operation.

This initial assessment showed that Caesarean sections can safely be done in rural areas of southern Ethiopia. The post-operative mortality was approximately 1%, which is acceptable by WHO targets [25].

### Estimating maternal mortality

We used the WHO-ICD-10 definition and classification of maternal deaths. Thus, if a mother died during pregnancy, during delivery, or within 42 days after the delivery or termination of pregnancy, we classified this death as a maternal death [26]. However, if the women died of an accident or suicide we did not label it as a maternal death. We also used the WHO maternal death review manual of 2004 to classify the possible causes of deaths [17]. Since this work was done in a rural setting with limited access to health institutions, the cause of death was based on our history taking of the symptoms such as convulsions, fevers or excessive bleeding due to haemorrhage.

### Data management and analysis

The setup of the birth registration system has been previously described [8]. In brief, we trained all health extension workers before starting the registration, and we employed supervisors who were trained nurses to visit each of the health posts. The data were entered in a printed registry book at each health post by the health extension workers. Each month, nursing supervisors visited the health post, checking the registry for completeness, supervising the health extension worker and taking a copy of the registry book to the project office.

Our birth registration contains about 3½ years of valid data. In late 2013, the birth registries were transferred to the local government system. This caused an interruption that resulted in data that could not be fully quality assured, so we have therefore only included data in which supervisory nurses checked the quality of the birth registration.

The availability of Emergency Obstetric Care is important to help avoid maternal deaths due to life-threatening complications [27]. The lack of transportation is a known barrier to the access of EmOC facilities, especially in rural areas [28]. In our study, we recorded both the type of road available close to the patient homes, assuming that road type is also associated with the availability of access to public transportation. We also recorded the walking time to the nearest institution in minutes. Unfortunately, the walking time in minutes showed large variances for the same locations; we therefore did not use it in our study. We used road access as a proxy measure for access to transportation, as has been validated by others [29].

Data were then entered into a computer using SPSS software (SPSS Inc., Chicago. IL), and the data were subsequently checked for completeness and errors, and the paper forms could be returned to the health post for further checks. For the analysis, we calculated proportions, incidence rates and odds ratios, and used logistic regression for multivariate analysis.

### Ethics statement

This is an implementation study, and the work done was a part of the routine work of the hospitals, health institutions and health extension workers in the area. For the research part, the Institution Review Board for Health Research of Southern Nations Nationalities and Peoples’ Regional State (SNNPRS) in Ethiopia, and the Regional Committee for Health Research Ethics of North Norway (REK Nord), approved the study.

## Results

### Some background information

The study covered all the areas of the selected woredas, and we registered 38,312 births: 52% boys and 48% girls. The mean age the mothers were 28.1 years (SD 4.4 years). 74% of the mothers were illiterate, and only 2% of the mothers had completed high school. Among the fathers, 53% were illiterate, and 5% had completed high school.

### Antenatal controls and referrals

The mean number of antenatal visits for each woman was 2.6 (interquartile range (IQR) 2–4). However, this varied between the districts with an average of 3.0 (IQR 2–4) visits in Dirashe, 2.2 (IQR 1–3) visits in Bonke and 2.7 (IQR 2–4) visits in Arba Minch Zuria.

Between 2010 and 2013, the percentage of pregnant women who attended four or more pregnancy controls improved by 28.4% in Dirashe (P<0.001), by 11.5% in Arba Minch (P< = 0.001) and by 16.5% in Bonke (P<0.001). Similarly, the number of women referred to an institution with a skilled birth attendant increased most in Dirashe, followed by Arba Minch, and least in Bonke (Table 2).

**Table 2:** Antenatal controls for each area and year.

Woreda	Year	More than four ANC visits		Three or less ANC visits		Total
**Dirashe**	2010	1,038	33.1	2,094	66.9	3,132
	2011	1,169	43.5	1,519	56.5	2,688
	2012	1,041	43.4	1,355	56.6	2,396
	2013	1,229	59.5	836	40.5	2,065
	Total	4,477		5,804		10,281
**Bonke**	2010	672	18.0	3,065	82.0	3,737
	2011	1,005	21.2	3,742	78.8	4,747
	2012	1,111	24.8	3,361	75.2	4,472
	2013	422	34.5	802	65.5	1,224
		3,210		10,970		14,180
**Arba Minch**	2010	1,147	27.0	3,105	73.0	4,252
	2011	1,513	33.5	3,010	66.5	4,523
	2012	1,266	37.9	2,078	62.1	3,344
	2013	666	38.5	1,066	61.5	1,732
		4,592		9,259		13,851
**Total**	2010	2,857	25.7	8,264	74.3	11,121
	2011	3,687	30.8	8,271	69.2	11,958
	2012	3,418	33.5	6,794	66.5	10,212
	2013	2,317	46.1	2,704	53.9	5,021
**Total**		12,279		26,033		38,312

### Place of deliveries

Table 3 shows that the number of women who delivered at home declined from 89.8% to 69.2%, a decline of 20.4% (P<0.001) over the project period. The decline was 24.9% and largest in areas with CEmOC providing institutions (36.3% in Dirashe, and 11.6% in Arba Minch), compared with only 7.4% in the BEmOC providing area of Bonke (P<0.001). Similarly, the number of deliveries at health posts, health centres and hospitals increased in all the districts. The increase in institutional deliveries was highest in districts with CEmOC services in Dirashe (19.5%; P<0.001)), and Arba Minch Zuria (8.3%; P<0.001), compared to the district with only BEmOC services, Bonke (3.5%; P<0.001).

**Table 3:** Location of delivery.

Woreda	Year	Home delivery		Delivery at health post		Delivery at health centre		Delivery at hospital		Total
**Dirashe**	2010	2,835	90.5	43	1.4	26	0.8	228	7.3	3,132
	2011	2,372	88.2	78	2.9	26	1.0	212	7.9	2,688
	2012	1,890	78.9	198	8.3	153	6.4	155	6.5	2,396
	2013	1,119	54.2	376	18.2	365	17.7	205	9.9	2,065
		8,216		695		570		800		10,281
**Bonke**	2010	3,338	89.3	295	7.9	80	2.1	24	0.6	3,737
	2011	4,125	86.9	481	10.1	121	2.5	20	0.4	4,747
	2012	3,935	88.0	395	8.8	124	2.8	18	0.4	4,472
	2013	1,003	81.9	145	11.8	55	4.5	21	1.7	1,224
		12,401		1,316		380		83		14,180
**Arba Minch**	2010	3,812	89.7	97	2.3	176	4.1	167	3.9	4,252
	2011	3,989	88.2	142	3.1	230	5.1	162	3.6	4,523
	2012	2,767	82.7	136	4.1	257	7.7	184	5.5	3,344
	2013	1,355	78.2	93	5.4	200	11.5	84	4.8	1,732
		11,923		468		863		597		13,851
**Total**	2010	9,985	89.8	435	3.9	282	2.5	419	3.8	11,121
	2011	10,486	87.7	701	5.9	377	3.2	394	3.3	11,958
	2012	8,592	84.1	729	7.1	534	5.2	357	3.5	10,212
	2013	3,477	69.2	614	12.2	620	12.3	310	6.2	5,021
**Total**		32,540		2,479		1,813		1,480		38,312

Deliveries taken place at health centres and hospitals were carried out by skilled birth attendants.

### Referrals

The number of women referred to hospitals increased by 3.3% (P<0.001), and to health centres by 7.2% (P<0.001) (Table 4). The number of referrals increased in all areas, but more in CEmOC areas (from 8.6% to 26.3%; p<0.001) than in the area with BEmOC function (from 3.4 to 5.2%; p = 0.004).

**Table 4:** Referrals for each area and year.

Woreda	Year	Not referred		Referred to HC		Referred to Hospital		Total
**Dirashe**	2010	2,864	91.5	46	1.5	221	7.1	3,131
	2011	2,453	91.3	34	1.3	201	7.5	2,688
	2012	2,156	90.0	104	4.3	136	5.7	2,396
	2013	1,582	76.6	251	12.2	232	11.2	2,065
		9,055		435		790		10,280
**Bonke**	2010	3,614	96.7	95	2.5	28	0.7	3,737
	2011	4,591	96.7	128	2.7	28	0.6	4,747
	2012	4,345	97.2	103	2.3	24	0.5	4,472
	2013	1,164	95.1	35	2.9	25	2.0	1,224
		13,714		361		105		14,180
**Arba Minch**	2010	3,926	92.5	165	3.9	154	3.6	4,245
	2011	4,216	93.2	150	3.3	156	3.4	4,522
	2012	2,857	855	309	9.2	175	5.2	3,341
	2013	1,375	82.3	211	12.6	84	5.0	1,670
		12,374		835		569		13,778
**Total**	2010	10,404	93.6	306	2.8	403	3.6	11,113
	2011	11,260	94.2	312	2.6	385	3.2	11,957
	2012	9,358	91.7	516	5.1	335	3.3	10,209
	2013	4,121	83.1	497	10.0	341	6.9	4,959
**Total**		35,143		1,631		1,464		38,238

### Birth attendants

We observed a decline in the use of traditional birth attendants over the period of intervention (Table 5). The decline was most marked in Dirashe (from 77% to 45%; P<0.001), followed by Arba Minch Zuria (31% to 15%; P<0.001) and less in the Bonke area, with BEmOC function only (from 29% to 25%; P<0.01). The use of relatives to assist deliveries remained fairly constant in all areas, whereas the use of health extension workers increased by 12.8% in Dirashe (P<0.001), 10.0% in Arba Minch Zuria (P<0.001), and was similar in Bonke with a 1.4% difference (P = 0.15). For health professionals (skilled birth attendants), the increase was 19% in Dirashe (P<0.001), 8.3% in Arba Minch Zuria (P<0.001) and 3.4% in Bonke (P<0.01), and most markedly in areas offering CEmOC services.

**Table 5:** The use of traditional birth attendants, health extension workers and health professionals for each area and year.

Woreda	Year	TBA		Relatives		Health Extension worker		Health professional		Total
**Dirashe**	2010	2,419	77.2	231	7.4	228	7.3	254	8.1	3,132
	2011	2,089	77.7	140	5.2	221	8.2	238	8.9	2,688
	2012	1,637	68.3	138	5.8	313	13.1	308	12.9	2,396
	2013	921	44.6	158	7.7	416	20.1	570	27.6	2,065
		7,066		667		1,178		1,370		10,281
**Bonke**	2010	1,101	29.5	1,839	49.2	693	18.5	104	2.8	3,737
	2011	1,391	29.3	2,344	49.4	871	18.3	141	3.0	4,747
	2012	1,303	29.1	2,199	49.2	828	18.5	142	3.2	4,472
	2013	301	24.6	603	49.3	244	19.9	76	6.2	1,224
		4,096		6,985		2,636		463		14,180
**Arba Minch**	2010	1,332	31.3	1,930	45.4	647	15.2	343	8.1	4,252
	2011	1,341	29.6	1,888	41.7	902	19.9	392	8.7	4,523
	2012	677	20.2	1,349	40.3	877	26.2	441	13.2	3,344
	2013	265	15.3	746	43.1	437	25.2	284	16.4	1,732
		3,615		5,913		2,863		1,460		13,851
**Total**	2010	4,852	43.6	4,000	36.0	1,568	14.1	701	6.3	11,121
	2011	4,821	40.3	4,372	36.6	1,994	16.7	771	6.4	11,958
	2012	3,617	35.4	3,686	36.1	2,018	19.	891	8.7	10,212
	2013	1,487	29.6	1,507	30.0	1,097	21.8	930	18.5	5,021
**SUM**		14,777		13,565		6,677		3,293		38,312

TBA: Traditional birth attendant. Only health professionals are skilled birth attendants.

### Maternal mortality rates

The maternal mortality rates declined by 64% during the intervention period, from 477 to 219 deaths per 100,000 live births (OR 0.46; 95% CI 0.24–0.88) (Table 6). The decline in MMR was higher for the woredas with CEmOC functions, Dirashe (67%) and Arba Minch Zuria (63%), than in the Bonke Woreda with BEmOC function only (32%).

**Table 6:** Maternal mortality rates (deaths per 100,000 live births) per woreda and year.

Woreda	Year	Maternal deaths	Number of deliveries	MMR
**Dirashe**	2010	14	3,132	447
	2011	3	2,688	112
	2012	4	2,396	167
	2013	3	2,065	145
		24	10,281	233
**Bonke**	2010	18	3,737	482
	2011	12	4,747	253
	2012	16	4,472	358
	2013	4	1,224	327
		50	14,180	353
**Arba Minch**	2010	21	4,252	494
	2011	16	4,523	354
	2012	7	3,344	209
	2013	4	1,732	231
		48	13,851	347
**Total**	2010	53	11,121	477
	2011	31	11,958	259
	2012	27	10,212	264
	2013	11	5,021	219
**Total**		122	38,312	318

MMR: Maternal Mortality rate (maternal deaths per 100.000 live births)

Almost 10% of the women reported to be ill during their pregnancy, during delivery or after delivery. Compared with the MMR of mothers who did not report illness or problems during pregnancy (MMR 116), the MMR among those who reported illness during the pregnancy MMR was 368 (OR 3.18; 95% CI 1.48–6.83), while for those who reported problems during and after delivery it was 2304 (OR 20.36; 95% CI 13.77–30.10). The MMR for women who reported problems during and after delivery declined from 2,984 in 2010 to 1,777 deaths per 100,000 live births in 2013.

During the period from 2010 to 2013, the MMR among the primigravidae was 490, for gravidae 2–5 it was 241 (OR 0.57; 95% CI 0.31–0.75) compared to the primigravidae, and among gravidae six or more, the MMR was 374 (OR 0.87; 95% CI 0.52–1.41). Over the years, the MMR declined in all groups, but most for the primigravidae from 913 in 2010 to 530 in 2013.

### Road access

As measured by the availability of road types, families with improved transportation to health services also had lower maternal mortality rates (Table 7). Households living close to all-weather roads had lower maternal mortality rates (MMR 220) compared with households living close to dry weather gravel roads (MMR 348; OR 1.58 (95% CI 2.05–2.38)) and those without roads (MMR 598; OR 2.72 (95% CI 1.61–4.61)) (Table 7).

**Table 7:** Mortality deaths a year road access to health institution.

Type of road		Number maternal deaths	MMR	Total
**Asphalt**	2010	5	432	1,157
	2011	3	196	1,530
	2012	1	85	1,181
	2013	0	0	592
	Total	9	202	4,460
**All-weather gravel**	2010	12	385	3,113
	2011	6	185	3,235
	2012	7	214	3,278
	2013	2	88	2,270
	Total	27	227	11,896
**Dry-weather gravel**	2010	24	441	5,445
	2011	17	282	6,020
	2012	14	286	4,893
	2013	8	456	1,753
	Total	63	348	18,111
**No driveable road**	2010	12	853	1,406
	2011	5	426	1,173
	2012	5	581	860
	2013	1	246	406
	Total	23	598	3,845
**Total**	2010	53	477	11,121
	2011	31	259	11,958
	2012	27	264	10,212
	2013	11	219	5,021
	Total	122	318	38,312

MMR: Maternal Mortality rate (maternal deaths per 100,000 live births).

In the results listed above, we have observed declines in maternal mortality rates in all areas. In parallel with the reduction in the maternal mortality rates, we observed significant changes in the use of antenatal services, increase in referrals to health institutions and a decline in the use of traditional birth attendants.

Women who attended four or more ANC visits had a lower risk of dying (MMR 204; OR 0.55, 95% CI 0.35–0.5) compared with women who attended three or less antenatal controls. Among deliveries with complications, we observed higher mortality rates among delivery cases referred to hospitals (MMR 1776; OR 4.02 (95% CI 2.31–6.99) and to health centres (MMR 920; OR 7.83 (95% CI 5.02–12.21)) than among deliveries that were not referred (MMR 230 deaths per 100,000 live births). Similarly, the MMR was higher if the delivery was attended by a skilled birth attendant (MMR 607; OR 2.09 (95% CI 1.29–3.36)). When doing a stratified analysis for the variables listed above, the association became non-significant for the years of 2012 and 2013.

In the logistic regression analysis, higher maternal mortality death rates were observed during the early phase of the project period, and if women attended few antenatal clinics, the delivery was attended by a traditional attendant or family member, and if the household did not have road access throughout the year.

## Discussion

In a large population, we carried out a complex intervention with the aim of reducing maternal mortality. Our main focus was to strengthen intrapartum care. In parallel with this study, we established a community-based birth registration system that we validated and used to monitor our intervention. Our results show that it is possible to achieve large reductions in maternal mortality rates over a short period of time if well-known and tested interventions are introduced.

This study evaluated the effects of several and coordinated interventions to reduce maternal deaths. By using a community-based birth registration, we document that maternal mortality rates were reduced by approximately 60% over a four-year period. We believe the main reasons for this decline are a decentralization of comprehensive and basic obstetric care. Through these interventions, we recorded increased referral rates, a decline in the use of traditional birth attendants and a decline in home births, as the proportion of pregnant women attending antenatal care increased. Even if the results are encouraging, we also observed that mortality rates remain high in areas with poor access, and among the poorer segment of the population [30].

This study was done in a typically rural mountainous area of Southwest Ethiopia. It includes areas with good access to health institutions, and areas with a varying access to basic and comprehensive emergency obstetric care: moreover, our findings could be generalizable to similar areas in Ethiopia. A key component of this intervention was the training of non-clinicians to do C/S, and of midwives to improve their performance and skills in basic emergency obstetric care. The training of non-physician clinicians was closely monitored through a prospective recording of their complications, and their performance was similar to that observed in other countries in Africa [6]. In addition, our intervention was followed by a population-based registration of maternal deaths, which enabled us to focus on areas with less declines in maternal mortality rates. The decline in maternal mortality rate in the Bonke area was probably less because they did not have access to CEmOC services. One clear strength of our study was the regular supervision done by senior health professionals, as such quarterly visits could have helped to maintain and improve the quality of the services. Another strength of this intervention was that the interventions were followed by community-based birth registrations. Although birth registrations are difficult to carry out, we believe that the model we use can be implemented in other areas. We quality-assured the validity of the registration of maternal deaths by using several tools for such assessments [3, 8, 30, 31].

Our study is an evaluation of a comprehensive set of interventions, and did not have a control group. By design, it is an observational study, so therefore several confounding factors that we did not measure could have influenced our results. One such reason could be that the Ethiopian government put a great effort in promoting institutional deliveries, use of antenatal care and the importance of maternal health. Because our work was an integrated part of the public health-care system in the country, we believe that these national efforts helped the intervention, since our work was done in a conducive political environment, and the government’s positive attitude might have favourably influenced the results.

We believe it would have been ethically unacceptable to conduct a randomized controlled study. However, given the nature of the interventions, we could compare the MMR in areas where comprehensive emergency obstetric care was present, and in areas where this service was only available if the patient could be transported to the nearest institution in another district.

Our study also shows a decline in maternal mortality rates over time in all areas. This shows that providing CEmOC services near the home of patients is an important strategy to improve maternal deaths. Furthermore, our data are based on a community registration of births. Thus, even if the number of referral and institutional delivery rates were improving, in absolute numbers they were moderately low. Nonetheless, we consider that the maternal mortality estimates are accurate, irrespective of whether the patient was referred or not. Although many studies show that institutional delivery rates are associated with reduced maternal mortality rates, this is not always true, and probably depends on the quality of obstetric services provided. In our case, we believe that the institutional delivery rates were far too low as the maternal mortality rates in home deliveries remained high in areas where higher referrals occurred, and in pregnancies where we did not record any illness.

Our line of reasoning as to why we consider the reduction in maternal mortality rates to be a result of the interventions are based on applying some of the Austin Bradford Hill’s criteria for assessing possible causal associations [32]. We found that the effect size is considerable (OR = 0.46), and that our results were consistent across several of the subgroups studied. For example, when stratifying the results for the use of antenatal services, education, referrals and transportation (road type), we see significant declines in maternal mortality rates in all the subgroups over the study period. The assessment of maternal mortality decline (effect measure) occurred after the intervention had taken place, thereby showing temporality between intervention and the expected outcome of the study. In our results, as has been reported by others, we also assessed a type of dose response relationship with, for instance, the use of transportation, where pregnant women with the least access to transportation had the highest maternal mortality rates compared to the groups with the best access to transportation [33]. And over the years, there has been a consistent decline in MMR for all these transportation groups.

Although there were few comprehensive studies to compare our results with, our results are in agreement with other studies that show associations between similar interventions and lower levels of maternal mortality [19, 34]. However, there are very few studies that have shown these associations in the same populations over time. Thus, we believe it is reasonable to conclude that the interventions played a part in reducing the maternal mortality rates.

Our study does not include an assessment of the costs of this programme. However, the external project costs were related to training, equipping institutions and supervising each of the institutions. The institutions had to cover the running costs, including salaries, routine expenses such as medicines and suture materials, as well as other costs for running the institutions. We therefore believe that the work at each institution will be sustainable provided the government can provide newly trained staff when such a need arises.

The intervention programme included all institutions and all communities (kebeles) in the area. However, in a mountainous area such as Southwest Ethiopia, we are aware that access and hence effective coverage were not equal. In a parallel study, we measured that the coverage of the work of the health extension workers is roughly 85%, with MMR slightly higher in areas not covered by the birth registration [8]. In the current study, we have also shown that people with less access to transportation had higher maternal mortality rates. Nevertheless, even if people lived in remote areas, we observed a decline in maternal mortality at all locations. A major challenge in the continuation of such interventions is to improve access by, e.g. the use of ambulances, including motorcycle ambulances that we later introduced into our programme.

Many studies have pointed to the lack of health-care seeking for ill mothers as risk factors for high mortality rates [35]. A recent meta-analysis documented that community-based interventions such as home visits and counselling can reduce foetal and neonatal mortality in low- and middle-income countries, whereas the effect on the reduction of maternal mortality was questionable [36].

Our study used a wide approach that included strengthening institutions, enhancing the referral system and strengthening the community work through the work of the health extension workers. Our aim was to decentralize services, and this integrated approach may have resulted in a large reduction in maternal mortality rates. Our results are therefore in agreement with a study from Tanzania, which showed that reduced access to a hospital can contribute to high levels of direct obstetric mortality [37].

A high pregnancy-related mortality among people living close to a hospital can suggest inadequate obstetric care at that hospital. Our study shows that the MMR declined more in areas with good access and with CEmOC, thus suggesting that the quality of care at such institutions had improved [37]. In countries with low-quality health-care systems, women in need of delivery services who are referred to an institution can experience higher case fatality rates (number of maternal deaths in an institution divided by the number of deliveries in the same institution) than population-based MMR estimates [3, 38–40]. Possible causes for the high institutional case fatality rates include a low quality of emergency obstetric care, including unnecessary delays at the institutions [41, 42]. During the first year of our study, we observed a higher MMR among those who were referred, but this association became non-significant, and that could mean that the quality of the healthcare at the institutions had improved.

One controversy in health-care delivery in rural Africa has been the role of traditional birth attendants in improving delivery services [43]. Although we did not aim to reduce the role of traditional birth attendants, we noted a substantial decrease in the use of TBAs, and also that such a decline was associated with a reduced MMR. We interpret these changes in that improved quality through the public health-care system replaced the use of traditional birth attendants. However, the Dirashe, Arba Minch and Bonke woredas differ in their use of traditional birth attendants. The latter two woredas belong to a similar language group and with similar cultural norms, while Dirashe is linguistically and culturally different.

Because this was an intervention carried out by the public health institutions, and the project was in line with established government policies, the acceptability of the interventions was very good and supported by local and regional health authorities. Such an approach also assures that the project is adapted to the local needs, and that the population accepts the interventions as appropriate.
